# Ecology and responses to climate change of biocrust-forming mosses in drylands

**DOI:** 10.1093/jxb/erac183

**Published:** 2022-05-12

**Authors:** Mónica Ladrón de Guevara, Fernando T Maestre

**Affiliations:** Departamento de Desertificación y Geoecología. Estación Experimental de Zonas Áridas (CSIC), Carretera de Sacramento, s/n, 04120 La Cañada de San Urbano-Almería, Spain; Instituto Multidisciplinar para el Estudio del Medio ‘Ramón Margalef’, Universidad de Alicante, Carretera de San Vicente del Raspeig s/n, 03690 San Vicente del Raspeig, Spain; Departamento de Ecología, Universidad de Alicante, Carretera de San Vicente del Raspeig s/n, 03690 San Vicente del Raspeig, Spain; University of Parma, Italy

**Keywords:** Abiotic interactions, biological soil crusts, biotic interactions, bryophytes, global change, hydrology, microbial community, nutrient cycles, plant interactions, soil properties

## Abstract

Interest in understanding the role of biocrusts as ecosystem engineers in drylands has substantially increased during the past two decades. Mosses are a major component of biocrusts and dominate their late successional stages. In general, their impacts on most ecosystem functions are greater than those of early-stage biocrust constituents. However, it is common to find contradictory results regarding how moss interactions with different biotic and abiotic factors affect ecosystem processes. This review aims to (i) describe the adaptations and environmental constraints of biocrust-forming mosses in drylands, (ii) identify their primary ecological roles in these ecosystems, and (iii) synthesize their responses to climate change. We emphasize the importance of interactions between specific functional traits of mosses (e.g. height, radiation reflectance, morphology, and shoot densities) and both the environment (e.g. climate, topography, and soil properties) and other organisms to understand their ecological roles and responses to climate change. We also highlight key areas that should be researched in the future to fill essential gaps in our understanding of the ecology and the responses to ongoing climate change of biocrust-forming mosses. These include a better understanding of intra- and interspecific interactions and mechanisms driving mosses’ carbon balance during desiccation–rehydration cycles.

## Teaser

This review synthesizes our understanding of key topics related to the ecology of biocrust-forming mosses in drylands and the plausible effects of climate change on them.

## Introduction

Drylands represent the largest terrestrial biome, occupying ~ 41% of the global land area (Cherlet *et al.*, 2018). A heterogeneous cover composed of patches of vascular plants surrounded by rocks and bare or biocrust-dominated soils characterizes these water-limited landscapes (Viles, 2008). Biocrusts, diverse communities of organisms (heterotrophic and photoautotrophic bacteria, archaea, protists, algae, fungi, lichens, mosses, liverworts, nematodes, microarthropods) living within the first centimetres of the soil surface (Weber *et al.*, 2016), constitute a significant feature of stressful environments such as drylands. These communities, whose global coverage is estimated at around 12% of Earth’s terrestrial surface (Rodriguez-Caballero *et al.*, 2018), can dominate the plant interspaces in many drylands due to their specific adaptations to cope with high insolation, low rainfall, and drought. In these systems, the species richness of mosses is low compared with other wetter regions: only about 250 of the approximately 11 000 species of mosses known have been recorded as a biocrust component, and most of these species are from the families Pottiaceae, Grimmiaceae, and Bryaceae (Zhao *et al.*, 2009; Geffert *et al.*, 2013; Seppelt *et al.*, 2016). However, biocrust-forming mosses are common in drylands worldwide (Bowker *et al.*, 2016; Maestre *et al.*, 2021), where they typically form part of late-successional biocrusts (Belnap and Eldridge, 2003, Deng *et al.*, 2020).

During the past three decades, a relevant body of literature underpinning the importance of vascular plant diversity for ecosystem functions and services has emerged (e.g. Hector *et al.*, 2010; Liang *et al.*, 2016; Duffy *et al.*, 2017). Mosses also provide critical ecosystem services, but their study has largely been ignored until recently (Cornelissen *et al.*, 2007). In drylands, mosses can act as ecosystem engineers regulating soil properties, microbial communities, and key ecosystem processes such as infiltration, nutrient cycling, and carbon (C) sequestration (Bowker *et al.*, 2011; Delgado-Baquerizo *et al.*, 2016, 2018; Bao *et al.*, 2019). In addition, they can also promote the establishment of vascular vegetation during ecosystem restoration (Havrilla *et al.*, 2019, 2020; Chen *et al.*, 2020). However, it is also possible to find contradictory information about their effects on ecosystem processes and interactions with other organisms. Several reviews and meta-analyses have attempted to clarify better the ecological roles of biocrusts. Recent reviews have focused on their influence on the hydrological (Belnap, 2006; Eldridge *et al.*, 2020) and nutrient (Barger *et al.*, 2016; Sancho *et al.*, 2016) cycles, their roles as soil stabilizers (Belnap and Büdel, 2016), their interactions with vascular plants (Y. Zhang *et al.*, 2016; Havrilla *et al.*, 2019), or their physiological ecology (Coe *et al.*, 2014). However, most of these reviews are not specifically focused on mosses, and their main goals are often to find general patterns without considering the importance of local environmental factors, linkages with other ecosystem processes, or species-specific traits. Besides, as in most organisms, an alteration of the current distribution of mosses is expected under future climate scenarios (Coe *et al.*, 2014, Rodriguez-Caballero *et al.*, 2018). To contribute to filling these gaps in the literature, here we review the adaptations of biocrust-forming mosses to drylands, their ecological roles in these ecosystems, and the potential impacts of climate change on these organisms and the ecosystem processes that rely on them.

## Adaptations of biocrust-forming mosses to dryland environments and their main biotic and abiotic constraints

Moss species are typically linked to humid habitats (Geffert *et al.*, 2013). However, a smaller group of species can thrive in harsh environments such as drylands. To do so, they have developed a unique variety of physiological and morphological strategies that allow them to survive in extreme habitats such as the Sahara, Mojave, or Atacama deserts (Ros *et al.*, 1999; Stark and Whittemore, 2000; Warren-Rhodes *et al.*, 2007). Water is the primary limiting factor for plant growth in drylands worldwide. Still, desiccation tolerance, i.e. the ability to dry to equilibrium with moderate to extremely dry air and to recover the normal metabolic functions after rehydration (Alpert, 2005), is relatively common in dryland mosses (Alpert and Oliver, 2002; Oliver *et al.*, 2005; Wood, 2007; Zhao *et al.*, 2015). Mosses have a maximal water content higher than other poikilohydric organisms such as lichens, which implies longer hydration periods after receiving water pulses (from rain, snow, dew, or fog) and the possibility of gaining more C through photosynthesis (Green *et al.*, 2011). However, they also have higher respiration rates and a lower ability to fix C below their optimum water content (Green *et al.*, 2011). These physiological traits can be a double-edged sword, and the result of having a positive C balance is highly determined by the frequency and magnitude of the water pulses that shape the desiccation–rehydration cycles (Coe *et al.*, 2014). In addition, and unlike green algal lichens that can reach positive net photosynthesis when the relative humidity is near saturation, most mosses rely on the presence of liquid water for activating their photosynthetic machinery (Rundel and Lange, 1980; Green and Lange, 1995). To mitigate these handicaps, mosses have physiological and morphological traits to take advantage of non-rainfall water. For example, their exposure to high atmospheric relative humidity prior to inputs of liquid water has positive effects on the recovery of the photosynthetic apparatus (Slate *et al.*, 2020a). Also, their leaves, which typically have recurved margins, papillose surfaces, and tips with excurrent hair-points (Longton, 1988), can act as condensation points of water vapour and divert the water toward the shoot apex or leaf itself, where it can finally provide metabolic activation and maintenance. This phenomenon has been recently described in the desert moss *Syntrichia caninervis*, which has a hierarchical water collection and storage system that comprises multiscale structures in the hairs for maximizing the exploitation of water inputs derived from dew, fog, and rainfall (Tao and Zhang, 2012; Pan *et al.*, 2016). Morphological functional traits also operate at several scales (i.e. at the leaf, shoot, and clump level) in bryophytes worldwide (Stanton and Coe, 2021). For example, Moore *et al.* (2016) found that the higher robusticity of shoots and taller clumps in female *Bryum argenteum* lend them greater water-holding capacity than male clumps.

**Fig. 1.**
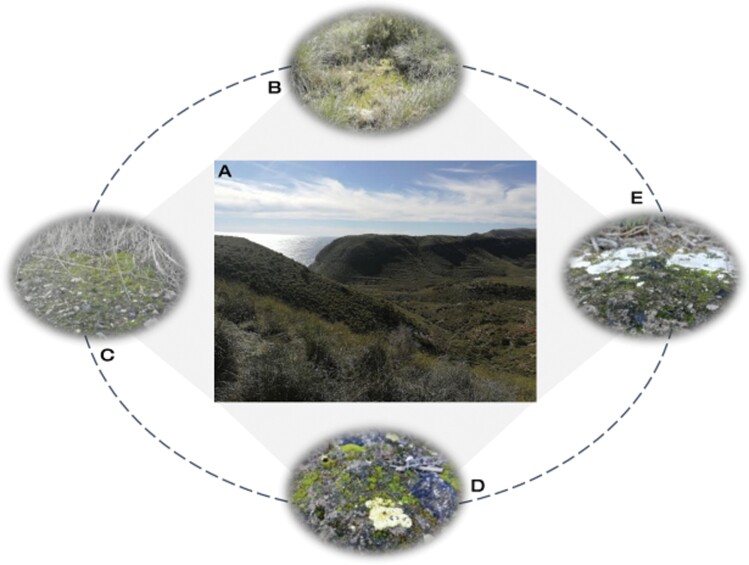
Example of distribution and biotic interactions of moss-dominated biocrusts in drylands. Cabo de Gata-Níjar Natural Park (A) is a Mediterranean coastal steppe ecosystem in southeast Spain and one of the driest sites in Europe. The long-term average rainfall is around 200 mm, but an important source of water for vegetation comes from fog and dew. This allows a well-developed grassland vegetation dominated by sparse tussocks of *Macrochloa tenacissima* with open spaces where mosses are abundant (B). A preferential microhabitat for biocrust-forming mosses is located under the canopy of these tussocks (C). In Mediterranean drylands is very common to find biocrusts where mosses coexist with lichens such as *Fulgensia* spp. (D) or *Squamarina lentigera* (E).

These physiological and morphological characteristics of biocrust-forming mosses largely determine their frequency and distribution patterns in drylands. In part due to their dependency on liquid water, their presence and abundance in hyperarid and arid habitats are lower than those of other biocrust constituents (Belnap *et al.*, 2001; Maestre *et al.*, 2021), and their clustered distribution increases proportionally with the aridity of the environment (Navas Romero *et al.*, 2020). This distribution indicates the requirement of an environmental niche narrower than that of other biocrust members for success in drylands, which is generally determined by higher soil moisture and shade levels. For this reason, mosses are more frequent on the north slopes of arid and semiarid landscapes (Nash *et al.*, 1977; Kidron, 2014a; Zhou *et al.*, 2020), and their richness and dominance within biocrust communities can significantly increase with precipitation (Li *et al.*, 2017). Also, when vascular plants colonize dryland areas, they usually create microhabitats more suitable for mosses than for lichens or cyanobacteria (Martínez *et al.*, 2006; Hernandez and Knudsen, 2012; Li *et al.*, 2017; Blanco-Sacristán *et al.*, 2021). Nevertheless, the preference for these microhabitats can differ among moss species due to their different adaptations to light intensity (Fig. 1). Those species that can take advantage of variable light and brief sun flecks for photosynthesis can also increase their hydration time as the surface evaporation is lower under the canopy of vascular plants than in open sites. This prolongation of hydration time is especially true when the vascular plant that provides shade does not exploit the subsurface water soil content, avoiding in this way direct competition with mosses for this resource. An example of this is the association of the moss *S. caninervis* with the predominant shrubs of dryland areas of North America, and its competitive relationship for surface water and space with the annual invasive species *Bromus rubens* (Bowker *et al.*, 2000; Stark *et al.*, 2005). However, in Mediterranean drylands, mosses are quite common under the canopy of the perennial grass *Macrochloa tenacissima*, a species with a shallow root system (Martínez-Sánchez *et al.*, 1994; Maestre *et al.*, 2001). Some shrub species could also negatively interact with bryophytes due to their high litterfall rates (Thompson *et al.*, 2005), although this effect is still unclear as another study found developed moss-dominated biocrusts in habitats with high litter coverage (Briggs and Morgan, 2008). Thus, the relations between vascular plants and biocrust-forming mosses driven by litter could be complex because litter cover may affect several microenvironmental variables such as light intensity, temperature, moisture, and soil nutrient status (Y. Zhang *et al.*, 2016). In other biocrust constituents, such as cyanobacteria, green algae, and lichens, the relationship is negative due to a burial effect (Szyja *et al.*, 2019). However, some species of mosses could have particular adaptations to cope with this adverse effect (e.g. high shade tolerance or greater capacity to grow through the litter layer) and to take advantage of the positive effects provided by litter (e.g. greater concentration of nutrients and lower soil water evaporation).

In drylands with cold winters, snow can also cover moss crusts for prolonged times. However, snow is a critical source of water for biocrust-forming mosses during the melting period in spring and forms a layer protecting from subfreezing temperatures in winter. For example, in the Gurbantunggut Desert (China), several positive effects of an increase in snowfall have been reported: it can reduce the oxidative, temperature, and desiccation stresses during winter and spring (Zhang and Zhang, 2020), and the greater water availability it provides when it melts enables higher growth from spring to early autumn (Zhao *et al.*, 2016). Hui *et al.* (2018) also observed a positive effect of a moderate increase in snow depth on the chlorophyll content and photochemical efficiency after an individual snow event.

It is also possible to find dryland mosses with preferences for open, sun-exposed spaces (Soliveres and Eldridge, 2020). They also possess some morphological and physiological strategies for confronting the challenge of receiving intense solar radiation, especially when they are dry and cannot dissipate energy through photosynthesis. For example, *S. caninervis* can rapidly adjust the leaf angles to minimize or maximize light interception depending on its hydration level by employing biophysical turgor-driven reversible changes led by strategically located leaf cells (Zheng *et al.*, 2011; Wu *et al.*, 2014). Also, the acclimation capacity of desert mosses to different degrees of UV radiation is remarkable. A major mechanism is the ability to adjust photoprotective compounds according to the risks of suffering light damage by using pigmentation plasticity (Ekwealor and Fisher, 2020). As UV stress increases, mosses can reduce the chlorophyll to total pigment content ratio and increase the levels of zeaxanthin (a potential antioxidant) and chlorophyll *a*:*b* and carotenoid:chlorophyll ratios (Hamerlynck *et al.*, 2002; Ekwealor *et al.*, 2021). In addition to their presence, the location of photoprotective compounds in the cells can be essential for their functionality. For example, *Ceratodon purpureus*, a cosmopolitan species that can persist and be dominant in arid ecosystems (Weber *et al.*, 2018), has a lower total quantity of photoprotective compounds than *Bryum pseudotriquetrum*, another cosmopolitan species more abundant in wetter areas (Clarke and Robinson, 2008). However, *Ceratodon* has its ultraviolet screening compounds mainly located in the cell wall rather than inside the cells, which is why it has greater UV tolerance (Clarke and Robinson, 2008). Another central photoprotective mechanism in plants is the dissipation of excess energy as heat through a set of processes known as non-photochemical quenching. The relative importance of this mechanism in mosses is not evident. However, a recent study suggests that desert mosses can undergo a sustained form of non-photochemical quenching, and its relaxation after hydration is the main modulator of photosynthetic recovery, rather than the repair of damaged or inactivated photosynthetic systems (Ekwealor *et al.*, 2021). Finally, the different photoprotective mechanisms found in bryophytes seem to be more effective as their ability to tolerate desiccation increases, with the result that some dryland mosses can withstand both stresses (Takács *et al.*, 1999).

Along with climatic determinants such as aridity, edaphic factors also influence the distribution of biocrusts (Bowker *et al.*, 2017). Climate and soil properties are closely linked in drylands; as an example, aridity is the main factor responsible for soil salinity in continental ecosystems (Mota *et al.*, 2011). One type of saline soil typical of drylands is gypsum soil, which covers large arid and semiarid regions worldwide (Herrero, 2004). The high levels of calcium (Ca) in the form of gypsum are not a limiting factor for the distribution of mosses, and a high taxonomic and functional richness of mosses has been reported on gypsum soils in the USA, Europe, and Australia (Salmerón *et al.*, 2011; Aleffi *et al.*, 2014; Seppelt *et al.*, 2016; Bowker *et al.*, 2017). Some specialized gypsum species (e.g. *S. caninervis* var. *gypsophila*, *Didymodon nevadensis*, and *Tortula revolvens*), strongly calcicolous species (e.g. *Aloina aloides*, *Crossidium crassinerve*, and *Weissia controversa*), and outcrop colonizers that can also be terricolous and require greater moisture and shade (e.g. *Gymnostomum calcareum*, *Eucladium verticillatum*, and *Pellia endiviifolia*; Aleffi *et al.*, 2014) appear to converge in gypsiferous areas. However, some species only reported in gypsum areas are at isolated sites and far from each other, meaning that gypseous substratum alone might not determine their colonization of new areas, but a set of microenvironmental conditions present in restricted sites within gypsum ecosystems (Guerra *et al.*, 1995; Aleffi *et al.*, 2014).

The fact that some of these species are also found on soils enriched with Ca carbonate (CaCO_3_) suggests that they require or tolerate high Ca levels. For example, the abundance of mosses has been related to high soil pH, electrical conductivity, and Ca levels in Australia (Downing and Selkirk, 1993). A certain degree of soil stability is also necessary for mosses, and soils with a finer texture, which provide an inherent stability and increase water retention, can favour their growth (Downing and Selkirk, 1993; Bowker *et al.*, 2006). In conclusion, the richness and diversity of biocrust-forming mosses in drylands are determined by a convergence of large- and local-scale environmental variables. Mosses are moisture-limited at a large scale (Concostrina-Zubiri *et al.*, 2014b), but the positive effect on water availability of biotic and abiotic variables at the microhabitat scale (e.g. soil texture and radiation interception by vascular plants and topography) and the physiological and morphological adaptations explained in this section, allow them to penetrate the more arid regions of the world.

## Biocrust-forming mosses as ecosystem engineers in drylands

### Building the foundations: effects of mosses on soil properties and their conservation

**Fig. 2.**
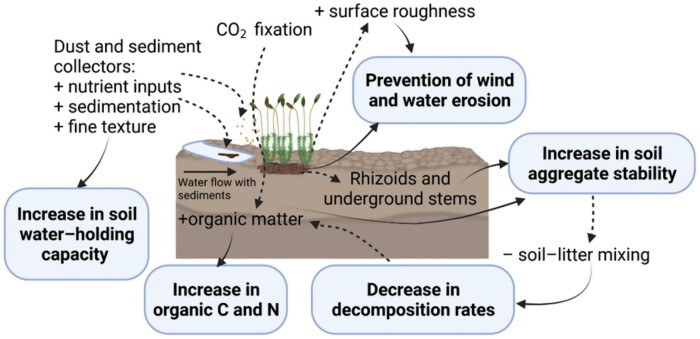
Effects of mosses on soil properties. Soil variables and processes increased and decreased by the presence of mosses are indicated by + and – signs, respectively. Created with BioRender.com.

Soil erosion is a primary determinant of land degradation and desertification in drylands (Kidane *et al.*, 2019; Chen *et al.*, 2021). Sediment capture by biocrusts is a key functional trait of these communities that directly influences their capacity to aggregate soil particles and thus control erosion (Mallen-Cooper and Eldridge, 2016). There is a strong consensus that biocrust-forming mosses prevent both wind and water erosion (Yang *et al.*, 2014; Bu *et al.*, 2015) and are more effective than other biocrust constituents in doing so (Mallen-Cooper and Eldridge, 2016; Gao *et al.*, 2020a, b). Their use to prevent erosion has even provided better results than those of vascular plants in some cases (Zhao and Xu, 2013; Wang *et al.*, 2021). Biocrusts can act against erosion through several mechanisms. The most evident is the physical barrier, but other physical properties, such as their effects on soil surface roughness, can also play a primary role in energy dispersion processes (Eldridge and Rosentreter, 1999; Wang *et al.*, 2017). However, the effectiveness of this property is intrinsically linked to water content. Many mosses shrivel, fold, or curl during dry periods, losing a relevant volume, but upon rehydration, they can rapidly recover their volume and increase soil roughness (Danin and Ganor, 1991; Warren, 2001). This wetting-induced roughness is much greater in mosses than in other biocrust constituents (Wang *et al.*, 2017) and can explain the high capacity of mosses to absorb raindrop and runoff kinetic energy, and thus to reduce erosion associated with rainfall splash and overland water flow (Kidron *et al.*, 2003; Y. Zhao *et al.*, 2014). Besides, the increase in surface roughness provided by mosses reduces wind speed and allows the capture of dust and nutrients (Danin and Ganor, 1991; Williams *et al.*, 2012, 2013). In addition to physical mechanisms, changes in soil properties induced by mosses, such as increases in soil organic matter, cohesion, and fine soil texture, also protect the soil against erosive forces (Gao *et al.*, 2020b; Fig. 2).

### Biocrust-forming mosses as modulators of biogeochemical cycles

**Fig. 3.**
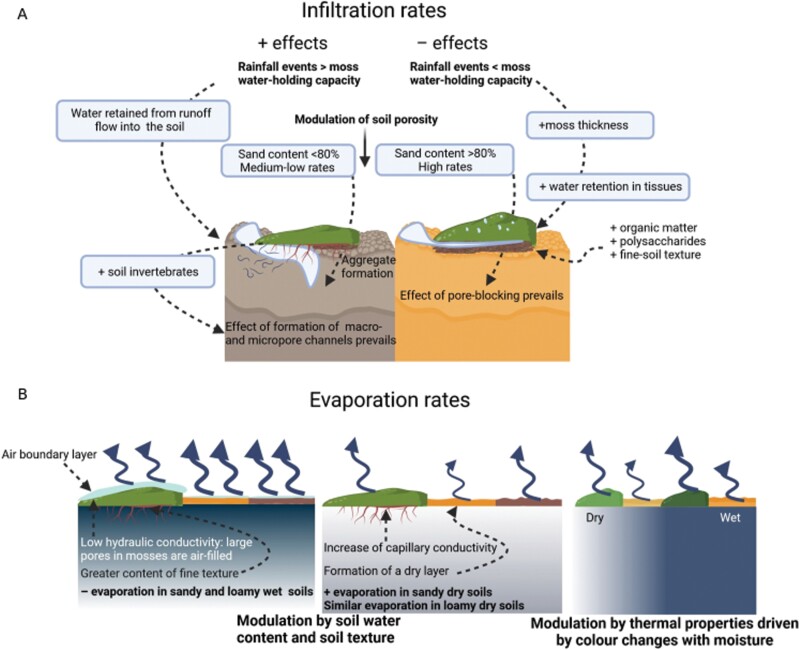
The effects of mosses on the soil water balance are determined by water infiltration into the soil (A) and evaporative processes (B). Positive signs on biotic and abiotic variables indicate their positive correlations with the presence of biocrust-forming mosses in (A). The increase of the variables located in the soil profile on the left positively affects the infiltration rate, whereas those located on the right have a negative effect on this rate. The representation of mosses in (B) varies in colour to indicate the effect of radiation reflectance on evaporation. Created with BioRender.com.

In general, biocrusts are crucial to soil functioning in drylands as they influence the concentration of elements essential for the metabolism of soil organisms and vascular plants (Moreno-Jiménez *et al.*, 2020). It is well known that plant growth and ecological processes in drylands are strongly constrained by water availability, and biocrusts can significantly influence the distribution and preservation of water throughout the soil profile (Eldridge *et al.*, 2020). Biocrust-forming mosses can modulate both horizontal (runoff) and vertical (infiltration and evaporation) fluxes of water as well as soil moisture and water holding capacity (Eldridge *et al.*, 2020). However, there are inconsistent results regarding the effects of biocrust-forming mosses on hydrological processes due to the interactions of species-specific traits with site-specific characteristics and legacies, such as soil texture, climate, or previous disturbances. Factors such as surface roughness (Fig. 3) enhance surface soil vapour sorption and deposition of non-rainfall water (Tao and Zhang, 2012; Pan *et al.*, 2016; S. Li *et al.*, 2021a, b). Hence, biocrust-forming mosses can increase soil moisture in the first centimetres but decrease it at deeper horizons in sandy soils (Yang *et al.*, 2014; Xiao *et al.*, 2016). These organisms can also prevent water losses through evaporation after rainfall events and, therefore, increase soil moisture during this time (Y.-F. Zhang *et al.*, 2016). However, hydric conditions can modulate this response since the capacity of mosses to mobilize water by capillarity has a negative relationship with their moisture level (Voortman *et al.*, 2014). Thus, the water that mosses retain by adsorption after a rain event can be easily lost 3 or 4 d after that, especially if the mosses are dark green coloured (Xiao *et al.*, 2010; Y.-F. Zhang *et al.*, 2016). Altogether, the final balance of moss effects on soil moisture depends on local features (e.g. characteristics of rainfall events and soil properties) and the traits of the moss species. This could explain results in which moss cover either decreased the soil moisture in the first layers of the soil (e.g. Bu *et al.*, 2015; Xiao and Hu, 2017) or enhanced it (e.g. Xiao *et al.*, 2015, 2016; F. Sun *et al.*, 2021a).

**Fig. 4.**
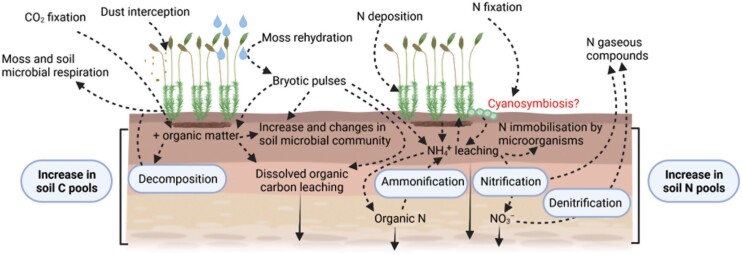
Effects of mosses on nutrient cycles. An interaction worth investigating in moss-dominated biocrusts is highlighted in red. Created with BioRender.com.

Just like vascular plants, mosses need nitrogen (N) for their growth. Uptake from wet or dry deposition has traditionally been considered their primary means of N acquisition (Brown and Bates, 1990). However, and despite a lack of developed roots and a vascular system, mosses can uptake N from soil and transport it to their shoots (Ayres *et al.*, 2006), and establish symbiotic associations with cyanobacteria, a group of N-fixer organisms, to obtain this macronutrient (Belnap, 2001). Cyanobacteria in soil crusts, as free-living organisms or as symbionts in lichens and mosses, significantly impact the N cycle as their N-fixing activity ranges from 40% to 85% of the total fixed biologically in terrestrial ecosystems at a global scale (Rodriguez-Caballero *et al.*, 2018). Unfortunately, there is still little known about the role of cyanosymbiosis in the nutrient status of moss-dominated biocrusts and how it interacts with soil N availability (Coe *et al.*, 2014). A recent study suggests that all these ways of acquiring N allow mosses to have enough N reserves even to transfer some of them to vascular plants through fungal loops without compromising their survival (Carvajal Janke and Coe, 2021). Mosses can also enrich the soil through direct N leakage during the cyanobacterial N fixation, the decomposition of moss tissues (Evans and Lange, 2001), or the phenomenon of ‘bryotic pulses’ (Slate *et al.*, 2019; Fig. 4). These pulses occur when mosses are rehydrated after a rainfall event and the cells damaged during the dehydration–rehydration process lose their intracellular content (carbohydrates, inorganic N, amino acids, and ionic compounds), which can ultimately be leached to the soil. Despite this, the effects of biocrust-forming mosses on the N cycle are poorly studied, particularly when compared with other biocrust constituents that are N-fixers (e.g. cyanobacteria or cyanolichens). The direction of N mobilization between the moss–soil interface is unclear and could depend on several biotic and abiotic factors. The population dynamics of the moss community could be one of them. In incipient communities, where mosses are colonizing new places and need to grow faster than their competitors for space, their N demand could be much higher than in stable communities. Thus, there is a mobilization of available N—ammonium (NH_4_^+^) or nitrate (NO_3_^−^)—from soil to mosses in these situations. This factor in the direction of N mobilization agrees with results from Slate *et al.* (2020b), who found that the establishment of a moss-dominated biocrust for 3 years after its inoculation caused a decrease of NH_4_^+^ concentration in the soil beneath. However, the NO_3_^−^ concentration in the soil was not affected by moss cover, also supporting the idea that NH_4_^+^ instead of NO_3_^−^ is energetically much more efficient for generating new moss biomass (Ruan and Giordano, 2017). An alternative explanation of the decrease of ammonium caused by mosses is that the ‘bryotic pulses’ enhance the microbiome responsible for N immobilization (Slate *et al.*, 2020b). However, more research is needed to elucidate the main mechanism of ammonium decrease in soils colonized by mosses. In mature communities, N can be transferred from mosses to the soil, and abiotic factors such as rainfall inputs drive the magnitude of this transfer (Slate *et al.*, 2021). Events causing high mortality of mosses can also significantly alter N pools in soils (Reed *et al.*, 2012). After these events, Reed *et al.* (2012) observed a switch from NH_4_^+^ to NO_3_^−^ dominance in a dryland ecosystem in Utah. This shift in the soil N pools has important implications for ecosystem functioning. On the one hand, although NO_3_^−^ is energetically less effective in plant nutrition, its greater mobility in most soils and lower use by microorganisms could increase its availability for plants (Austin *et al.*, 2006). But on the other hand, the easier loss of this N component through gaseous emissions (McCalley and Sparks, 2009; Weber *et al.*, 2015) could reduce its content in drylands.

Since N is a primary limiting element for C acquisition by plants, mosses can act as modulators of the C cycle through their impacts, as explained, on the different forms of N and their links with other organisms. Nevertheless, mosses also have a direct role by C fixation through photosynthesis. The functional traits that govern the C balance in biocrust-forming mosses are still not well understood. Different moss species have a broad range of responses to environmental conditions, especially to water input patterns (Coe *et al.*, 2019). Among the different biocrust constituents, mosses usually have the highest photosynthetic efficiency in optimal moisture conditions (Lan *et al.*, 2017). Therefore, they have a higher potential to sequester C (Yang *et al.*, 2019; Xu *et al.*, 2022). However, they also have greater physiological limitations than lichens as aridity increases. For example, mosses have lower net photosynthetic rates when annual precipitation is below 200 mm (Raggio *et al.*, 2018).

Recent studies have highlighted the relevant role of the microbial communities within or below biocrusts as modulators of nutrient cycling (Liu *et al.*, 2017; Concostrina-Zubiri *et al.*, 2021; Qi *et al.*, 2021). Specifically, biocrust-forming mosses can increase the abundance and diversity of bacteria and fungi beneath them (Liu *et al.*, 2018; Maier *et al.*, 2018). The fungi:bacteria ratio and the functional genes involved in C and N cycles also increase under moss-dominated biocrusts compared with other less-developed biocrust types (Liu *et al.*, 2018). These genes were linked to C degradation and N denitrification, causing, for example, an increase in respiration and nitrous oxide (N_2_O) emissions in moss-crusted soils (Maier *et al.*, 2018; Slate *et al.*, 2019). However, the nitric oxide (NO) and nitrous acid (HONO) effluxes in biocrust-forming mosses are lower than in cyanobacteria-dominated biocrusts (Weber *et al.*, 2015; Maier *et al.*, 2018). These different efflux dynamics reflect the necessity of studying multiple N compounds to unravel the role of mosses in N cycling. In conclusion, the net effect on soil nutrient content will be modulated by abiotic factors that drive processes such as leaching and microbial activity.

### Environmental gradients modulate biotic interactions involving mosses

Biocrust-forming mosses have a large capacity to alter the soil’s physical and chemical properties, and by doing so, mosses can affect the performance of vascular plants. However, the impacts of mosses on vascular plants are not clear-cut and seem to be strongly modulated by environmental gradients (Doxford *et al.*, 2013). A recent meta-analysis (Havrilla *et al.*, 2019) found that, in general, the positive effects of mosses on the performance of vascular plants (i.e. germination, survival, growth, cover, nutrient uptake, phenology, reproduction, and diversity) prevails over the negative ones. This result contrasted with the effects of cyanobacteria and lichens on vascular plants, as they came out as negative (Havrilla *et al.*, 2019). Biocrust-forming mosses can promote vascular plant growth by forming soil fertility islands (Ferrenberg *et al.*, 2018). As commented above, these nutrients can be mobilized from open spaces to vascular plants through fungal loops. However, it is also possible to find examples where mosses undermine vascular plant growth due to their competition for water and their capacity to reduce water infiltration through the soil profile (Guan and Liu, 2019; X. Li, Yu *et al.*, 2021).

**Fig. 5.**
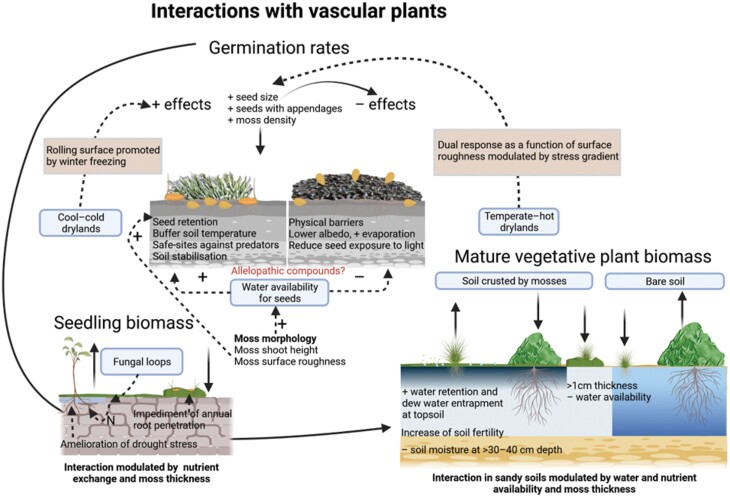
Different moss–vascular plant interactions. In the germination rates section, biotic factors with positive (+) signs and an arrow pointing to the soil profile on the left indicate positive correlations with germination rate, whereas those pointing to the right indicate negative correlations with this rate. An interaction worth investigating in moss-dominated biocrusts is highlighted in red. Created with BioRender.com.

This competition can be highly modulated by environmental conditions, especially precipitation. For example, in the Negev desert, the highest densities of vascular plants under near-average precipitation conditions can be found at the base of dunes. In these sites, moss-dominated biocrusts are dominant, and the water supply is higher than in the dunes themselves (Tielbörger, 1997). However, in years of extreme drought, water availability is higher in non-crusted mobile dunes, so the bloom of annual species takes place in these areas (Kidron, 2014b). The effect of mosses on the germination of vascular plants can range from positive to negative (e.g. Y. Zhang *et al.*, 2016; Ferrenberg *et al.*, 2018; Havrilla *et al.*, 2019; X. Li, Yu *et al.*, 2021), a range of responses likely driven by the species-specific requirements for seed germination. Mosses have a high capacity to modulate the soil microenvironment beneath them through their morphology and light reflectance. Consequently, the degree to which the optimum environment for seed germination meets the temperature and moisture ranges influenced by moss traits will determine the result of this moss–plant interaction (Fig. 5). For example, Schlatterer and Tisdale (1969) found a possible inhibitory effect of moss litter on seed germination in only one of three grass species tested. In some cases of very well-developed crusts, germination can be prevented because seeds do not reach the soil surface (McIlvanie, 1942). Thus, the morphology and dispersal mechanisms of seeds could also be crucial to ensure their germination within moss-dominated biocrusts.

Competition for space can act as a powerful structuring force of biocrust communities in drylands (Maestre *et al.*, 2008; Bowker *et al.*, 2010). Dominant moss species in these communities are often large-sized species with a high growth rate, as has been detected for the species *Pleurochaete squarrosa* across an environmental gradient in Spain (Bowker and Maestre, 2012). Those moss species are also more effective in the retention of water and the uptake of nutrients such as N and phosphorus (P) (Li *et al.*, 2019). One of their competitors for space, lichens, can produce around 800 different secondary compounds (Asplund and Wardle, 2017). Thus, it is not unreasonable to think that some of them can be used as chemical weapons against bryophytes. For example, Gardner and Mueller (1981) examined in the lab the toxicity potential of several lichen acids on the germination and sporeling development of *Funaria hygrometrica*. Some of them had negative effects on these parameters, although their relative toxicity was highly dependent on their concentration and the pH of the growing medium. Thus, it is still difficult to interpret the potential of the results in the real world. Also, a non-hierarchical competition (also called intransitive competition), where there are no clear dominant or winner species, can exist within the members of biocrust communities (Bowker and Maestre, 2012). Soliveres and Eldridge (2020) found a microenvironment modulation of intransitive competition between moss and lichen species since this mechanism of community assembly has a higher role under shrub canopies than in open areas. These situations allow greater richness within the biocrust community as no one member is displaced and each can coexist with others.

Several studies also provide evidence of positive interactions between mosses and other biocrust constituents. Using a culturing approach, Bowker and Antoninka (2016) found a higher cover increase in a combination of lichens and mosses than in their respective monocultures. Colesie *et al.* (2012) found that moss-associated thalli of *Peltigera rufescens* had a higher net photosynthetic rate, thallus thickness and growth rate than those growing in isolation, providing a clear example of facilitation between mosses and lichens. Facilitative interactions in dryland biocrust communities seem to be less relevant as aridity increases (Bowker *et al.*, 2010). This fact is likely explained by the effects of mosses on water availability. In environments with very few rainfall events that allow poikilohydric organisms to reach their water holding capacity, mosses usually compete strongly for water. But under wetter conditions, mosses promote the infiltration and retention of water that can be used more gradually through capillarity by their neighbours (Eldridge *et al.*, 2010). However, the switch between competitive and facilitative interactions is not linear throughout stress gradients, but rather follows a U-shaped curve, with the maximum competition levels at the extremes (J. Sun *et al.*, 2021b). This curvilinear relationship can be observed when the analysis of the interactions includes all the main biocrust constituents and a wide stress gradient. In the wettest situations, cyanobacteria and algae are strongly displaced by lichens and mosses, although the specific mechanisms that provide these competitive advantages are still unclear. Mosses also interact with other members of the microbial communities in soils. The positive effects of mosses on soil stability and organic matter promote favourable microhabitats for microbial communities (Bao *et al.*, 2019) and, therefore, boost the diversity of bacteria and fungi. This biodiversity is higher when compared with bare and cyanobacterial crusted soils (Maier *et al.*, 2018; Tian *et al.*, 2021). However, these studies did not find significant differences when they compared the moss microbial communities with those located beneath lichens.

### The fate of biocrust-forming mosses in a changing world

**Fig. 6.**
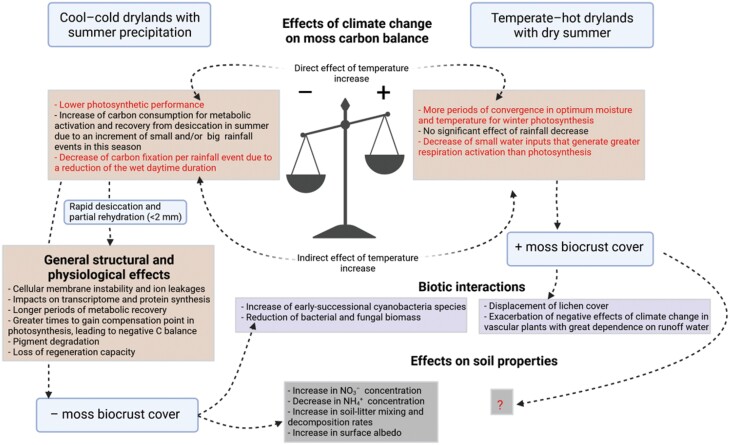
Loss of moss-dominated biocrusts driven by negative carbon balances in two climate change field experiments (Reed *et al.*, 2012; X. Li, Hui *et al.*, 2021) and in a mesocosm experiment (Tucker *et al.*, 2019) in cool drylands; and increasing (but not significant) trends of moss cover in two field experiments in temperate–hot drylands (Escolar *et al.*, 2012; Maestre *et al.*, 2015; Ladrón de Guevara *et al.*, 2018). Effects related to temperature are highlighted in red. Biotic and abiotic effects found or suggested in these experiments are shown. The effects of mosses on soil properties are still inconclusive in temperate–hot drylands as lichens are the dominant biocrust component of these studies. In addition, several physiological effects derived from the desiccation–rehydration cycles caused by the treatments are presented as complementary mechanisms that could explain the observed changes in moss cover. Created with BioRender.com.

The effects of global warming and altered rainfall regimes on biocrust-forming mosses are closely linked to their impacts on the water balance and desiccation–rehydration cycles of mosses. In those areas where large water inputs are dominant (versus non-rainfall water inputs such as fog and dew), a negative effect of elevated temperature through accelerated drying rates is expected in biocrusts dominated by mosses and lichens (Tucker *et al.*, 2019). However, a recent study using a long-term (53 years) record of biocrust surveys has found negative impacts of warming (~0.27 °C per decade) only in lichens, mosses being more sensitive to changes in precipitation (Finger-Higgens *et al.*, 2022). Mosses can fix more C than other biocrust types after large rainfall events. Still, they perform worst, and even have a negative C balance due to respiration, when subjected to small rainfall events (Zhang *et al.*, 2018) and/or prolonged desiccation periods (Coe *et al.*, 2012). If the pattern of small rainfall pulses during summer is prolonged over time, as several models forecast for some dryland regions (Miller *et al.*, 2021), this can lead to moss C starvation and a significant loss of their cover (Reed *et al.*, 2012; Ferrenberg *et al.*, 2015). The break of dormancy during summer, even with large rainfall events, can negatively impact on moss biomass (Stark *et al.*, 2011). However, warming can also reduce the frequency of very small events of water condensation and fasten the soil surface desiccation. These effects on water availability could also decrease the frequency of metabolic activations of mosses with a final negative C balance and shorten their respiration periods after photosynthetically unproductive small rainfalls (Fig. 6). In this case, an increase in temperatures can promote the development of moss-dominated biocrusts (Ladrón de Guevara *et al.*, 2018). Also, if the maximum photosynthetic period is determined by winter and spring rainfalls, an increase in temperature could favour a lengthening of the optimal temperature for photosynthesis in mosses, which ranges from 10 °C to 20 °C (Coe *et al.*, 2014). In fact, a favourable effect of spring precipitation on mosses was recently observed in a cool desert (Finger-Higgens *et al.*, 2022).

Bryophytes have evolved to use water when this resource is available aboveground (Proctor, 1982). It is thus reasonable to think that the desiccation–rehydration cycles that determine the C economy of mosses are more influenced by the frequency of water inputs than by their magnitude when the rainfall events surpass the water holding capacity of the upper soil layer. However, large rainfall events that cause water infiltration into deeper soil layers could positively affect the moss C balance as they could increase the period of optimum water content for photosynthesis through capillary processes (Ladrón de Guevara *et al.*, 2014). Although mosses in drylands are considered resistant to drought, multiyear drought events can overcome their resistance threshold and cause a population decline (Belnap *et al.*, 2006). Even for a single drought event, its duration can influence the speed of photosynthetic reactivation. This recovery speed could be key in determining the outcome of biotic interactions within the moss communities in regions such as the Mediterranean basin, as most climatic models converge in forecasting longer drought events in this area. For example, Munzi *et al.* (2019) found a slower photosynthetic reactivation in the dominant Mediterranean moss *P. squarrosa* after a 2-month drought than after a 2-week drought period. However, this effect was not observed in the alien moss *Campilopus introflexus*. Thus, climate change could also promote changes in the composition of moss communities in this area.

As mentioned above, seasonal snow can play a fundamental role in the physiological performance and biomass production of mosses in cold drylands. There is a consensus that the snowpack started to decline at the end of the last century along with an earlier spring melting in the northern hemisphere (Bormann *et al.*, 2018; Zhang and Ma, 2018). However, there is also a high regional variability, and in places where mosses are dominant, such as in the Gurbantunggut Desert, these trends are inverted (Tan *et al.*, 2019). In a scenario of a snow increase to twice that of the current regime, the physiological characteristics of mosses would allow them to increase their growth (Zhao *et al.*, 2016, 2018). Despite this, there are uncertainties about the impacts of snow changes due to its interaction with the expected increase in temperatures. For example, in the past decades, the trends of the snow/precipitation ratio modulated by temperature changes did not have the same direction across the Gurbantunggut Desert (Li *et al.*, 2018), and the forecasted trend in snow cover depth is a general decrease in almost all the area (Shi *et al.*, 2011). In this scenario, organisms with lower hydric requirements, such as cyanobacteria and algae, will likely displace mosses.

### Mosses as a tool for creating more resistant and resilient dryland ecosystems

Interest in using biocrusts for restoring ecosystem functions in drylands after relevant disturbance events has increased exponentially during the past decade. Biocrust-forming mosses have received particular attention because they usually have a greater impact on the ecological processes described above than the other biocrust members (Xiao *et al.*, 2019) and their *ex situ* culture for generating enough biomass for restoration work is feasible (e.g. Xu *et al.*, 2008; Y.-M. Zhao *et al.*, 2014; Antoninka *et al.*, 2016; Grover *et al.*, 2020). However, as this review has elucidated, mosses do not have a single effect on several ecosystem functions and it is important to consider site idiosyncrasy to predict restoration trajectories better. The level of degradation is an essential factor to consider when restoration goals are defined. When native populations persist in their natural locations, it is feasible to implement a passive recovery of moss populations in a time frame of 20 years, especially in grasslands and grassy woodlands with shaded areas where mosses have competitive advantages over other organisms (Read *et al.*, 2011; Concostrina-Zubiri *et al.*, 2014a). However, active restoration is required if the site has suffered an intense disturbance and there are not enough sources of propagules (Condon *et al.*, 2020). A first step for overcoming propagule limitations is the development of *ex situ* cultivation. For example, mosses grow well in trays with an organic substrate under greenhouse conditions (Grover *et al.*, 2020). Then, the translocation of the moss biomass obtained to the sites targeted for restoration is necessary and, nowadays, several techniques show promise for improving moss survival under field conditions in drylands (e.g. Blankenship *et al.*, 2020; Doherty *et al.*, 2020).

## Conclusions

Biocrust-forming mosses have relevant roles within and beyond biocrusts. Their distributions along dryland habitats are more constrained by environmental factors than those of early-stage biocrust constituents. However, they can displace other biocrust members when they successfully occupy spaces in their optimal environmental ranges. The distribution of mosses is more linked to vascular plants than that of lichens or cyanobacteria, especially when aridity increases. We have highlighted the critical role of biocrust-forming mosses in the hydrological cycle in drylands and in preventing soil loss, improving soil structure, and enhancing nutrient status in these areas. The interactions of biocrust-forming mosses with vascular plants are complex and, in most cases, species- and site-specific. The influence of mosses on the soil water content throughout the soil profile, and their radiation reflectance, morphology, and degree of development, can determine their effects on vascular plants. It is also difficult to forecast a general response of biocrust-forming mosses to climate change since the few existing field studies show divergent effects depending on local climatic characteristics. Some areas that require further research include a better understanding of the impacts of climate change on moss populations at the global scale, their biotic interactions within and outside biocrust communities, their effects on microbial communities and nutrient cycles, and the impacts of desiccation–rehydration cycles on their C economy. Identifying the best species to be used in restoration work and the best way of growing them are also key topics for future research.

## Data Availability

No new data have been analyzed or created in this review.
